# A new species of *Eriobotrya* (Rosaceae) from Yunnan Province, China

**DOI:** 10.3897/phytokeys.146.50728

**Published:** 2020-05-08

**Authors:** Su-Fang Chen, Kai-Kai Meng, Xi-Bing Guo, Wan-Yi Zhao, Wen-Bo Liao, Qiang Fan

**Affiliations:** 1 State Key Laboratory of Biocontrol and Guangdong Provincial Key Laboratory of Plant Resources, School of Life Sciences, Sun Yat-sen University, Guangzhou 510275, China Sun Yat-sen University Guangzhou China; 2 Malipo Laoshan Provincial Natural Reserve, Malipo 663600, China Malipo Laoshan Provincial Natural Reserve Malipo China

**Keywords:** chloroplast genome sequences, morphology, phylogeny, subtribe Malinae

## Abstract

*Eriobotrya laoshanica*, a new species of Rosaceae from Yunnan, China, is described and illustrated. The new species is easily distinguished from the most similar species *E. malipoensis* K. C. Kuan by its longer petioles (2–5 vs. 0.5–1 cm); indumentum on the lower leaf surfaces (densely tomentose vs. glabrous); much fewer flowers (15- to 30-flowered vs. 50- to 100-flowered) on the panicle; larger flowers (2.5–3 vs. 1.5–2 cm in diameter); and non-angulated (vs. angulated) young fruits.

## Introduction

The genus *Eriobotrya* Lindley, a small genus of subtribe Malinae (tribe Maleae, subfamily Amygdaloideae, Rosaceae) consisting of approximately 30 species, is distributed in Himalaya, eastern Asia and western Malesia (Vidal 1965; Gu and Spongberg 2003; Mabberley 2017). This genus is considered close to *Rhaphiolepis* based on the shared characters of larger seeds and thinner endocarp (Robertson et al. 1991). Recent studies based on molecular evidence strongly supported the *Eriobotrya*-*Rhaphiolepis* clade (Lo and Donoghue 2012; Xiang et al. 2016). *Eriobotrya japonica* (Thunberg) Lindley, commonly known as loquat, is an important fruit tree cultivated throughout southeastern Asia and southern Europe (Gu and Spongberg 2003).

There are about 16 *Eriobotrya* species (five endemic) recorded in China (Gu and Spongberg 2003; Yang and Lin 2007; Li et al. 2012). Among them, there are only four species and one natural hybrid species flowering in autumn and winter, namely, E. × daduheensis H. Z. Zhang ex W. B. Liao, Q. Fan et M. Y. Ding, *E. japonica*, *E. malipoensis* K. C. Kuan, *E. prinoides* Rehder et E. H. Wilson, and *E. serrata* J. E. Vidal (Gu and Spongberg 2003; Ding et al. 2015). In our investigations into *Eriobotrya* species in Yunnan province of China, a distinct *Eriobotrya* species flowering in autumn was collected in 2015. After four years’ field observations and comprehensive literature studies, we confirmed it was a new species and it is described and illustrated here.

## Materials and methods

Morphological observations of the putative new species and its close relatives were carried out based on living plants in the field as well as dried specimens. All morphological characters were measured using a stereomicroscope with ocular micrometer (Leica S8APO, Leica Microsystems Inc., Germany). The voucher specimens were deposited in the herbarium of Sun Yat-sen University (SYS) and the herbarium of South China Botanical Institute (IBSC).

Leaf samples for the putative new species were collected and stored in silica gel. The total DNA was extracted with the TIANamp Genomic DNA Kit [TIANGEN Biotech (Beijing) CO. Ltd] according to the protocol procedure, and then sent to Novogene Bioinformatics Technology (Beijing, China) Co. Ltd for quality inspection and low-coverage genome sequencing using Illumina 2000 platform following the standard Illumina sequencing procedure. Approximately 6 GB cleaned raw data was produced and assembled into circled chloroplast genomes with the perl script NOVOPlasty2.7.2 (Dierckxsens et al. 2017; accession numbers: MT130714, MT130715), using the chloroplast genome and the *rbcL* gene of *E. japonica* (downloaded from NCBI website, accession number: NC_034639.1) as reference and seed, respectively. Then the two assembled sequences were annotated on online GeSeq (Tillich et al. 2017) with the same reference of *E. japonica* (accession number: NC_034639.1). Further, complete chloroplast genome sequences for *Eriobotrya*, and other close genus such as *Rhaphiolepis*, *Heteromeles*, *Cotoneaster*, and *Photinia* were downloaded from the NCBI nucleotide database (Zhang et al. 2017). Together with the putative new species, all the chloroplast genomes were aligned with MAFFT version 7 (Rozewicki et al. 2019) and then manually checked and revised with MEGA version 6.0 (Tamura et al. 2013). The phylogenetic tree was then constructed with IQ-Tree 1.6.10 (Nguyen et al. 2015) based on the maximum likelihood method, in which the best-fit model of DNA substitution was auto-determined by calculating the Bayesian Information Criterion (BIC) using the 88 available nucleotide substitution models, ultrafast bootstrap was set as 2000, and *Photinia* species were set as outgroup.

## Results

### Molecular analyses

**Figure 1.**
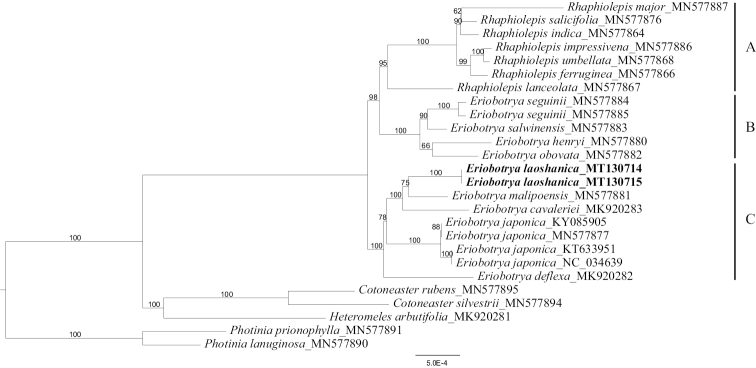
Maximum likelihood consensus tree of the new species and related species. Numbers above branches are ML bootstraps, the new species is shown in bold.

The alignment length of these twenty-five chloroplast genomes was 166,363bp in total, with the statistics of 1,307 parsimony-informative sites. No variable sites were detected between the two accessions of the new species but 139 variable sites were detected between the new species and *E. malipoensis*. This low diversity within the species was also observed in *E. japonica* (KT633951 was also identical to NC_034639, KY085905 identical to MN577877).The best-fit nucleotide substitution model was detected as TVM+F+R2 based on Bayesian Information Criterion (BIC). The ML phylogenetic tree (Fig. 1) showed that all the *Rhaphiolepis* species formed a well-supported clade (clade A) that is sister to a clade of *Eriobotry* species (clade B) and the sister group relationship of these two clades is well supported; *E. henryi*, *E. obovata*, *E. salwinensis* and *E. seguinii* clustered together forming the clade B; the clade A and B formed a sister relationship; the putative new species, *E. laoshanica* is placed into a well-supported clade with *E. malipoensis* and *E. calaleriei*, and then clustered with *E. japonica* and *E. deflexa* forming the clade C.

### Taxonomic treatment

#### Eriobotrya laoshanica

Taxon classificationPlantaeRosalesRosaceae

W.B. Liao, Q. Fan & S.F. Chen sp. nov.

49E803E9-0613-518F-A83F-0DF4012A1FB6

urn:lsid:ipni.org:names:77209566-1

##### Type.

**Figure 2.**
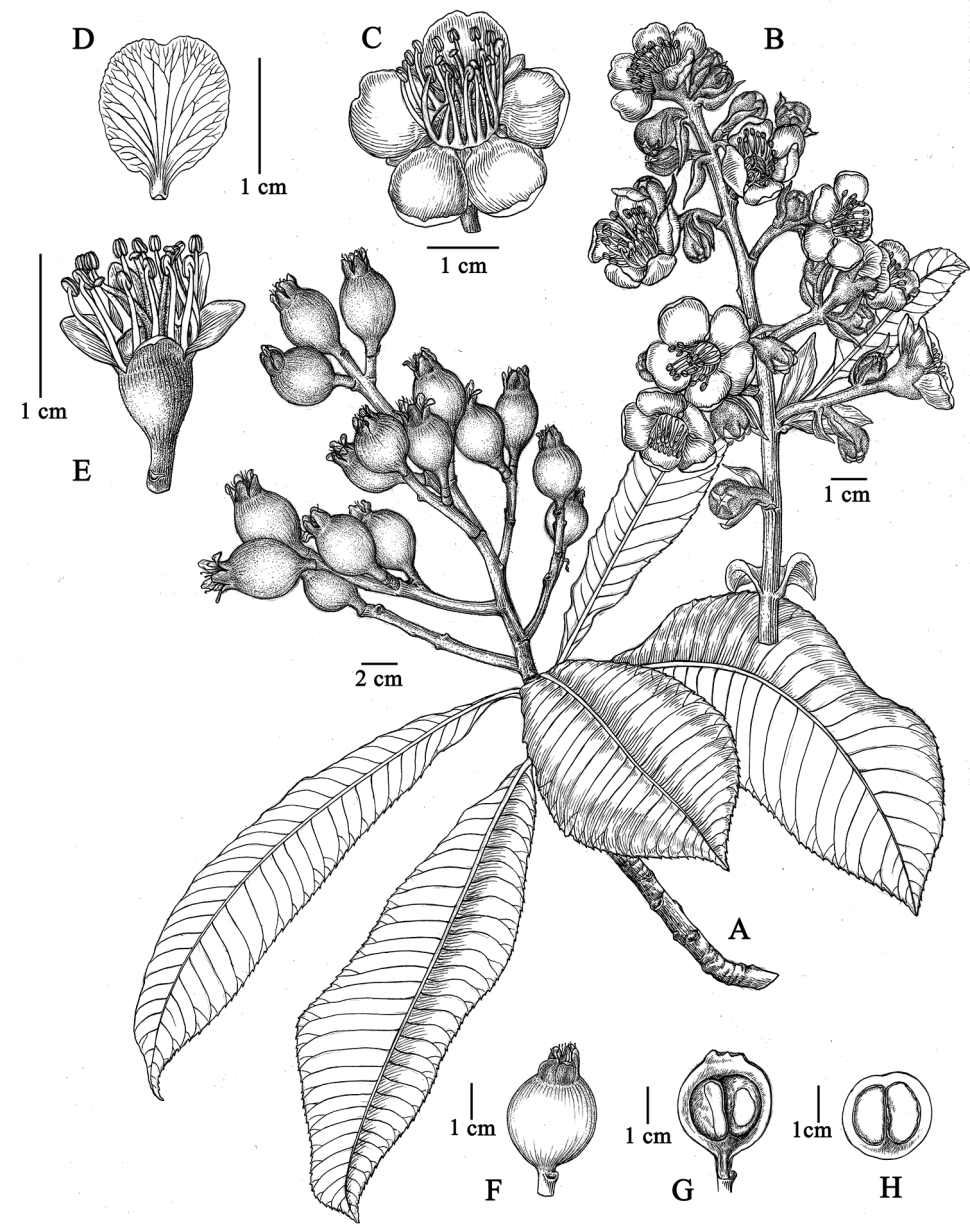
*Eriobotrya laoshanica* sp. nov. **A** fruiting branch **B** inﬂorescence **C** ﬂower, front view **D** petal, adaxial view **E** ﬂower without corolla showing hypanthium and calyx lobes **F** fruit **G** fruit, in longitudinal section **H** fruit, in transverse section. **A** and **F–H** from *Q. Fan 13900***B–E** from *Q. Fan 17570*. Drawn by Yun-Xiao Liu.

**Figure 3.**
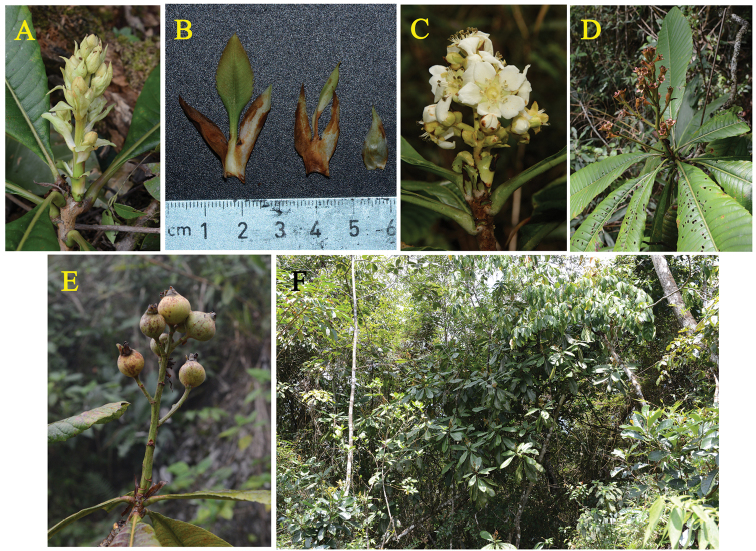
*Eriobotrya laoshanica* sp. nov. **A** young flowering branch **B** reduced leaves and bract **C** flowering branch **D** young fruiting branch **E** fruits **F** habitat.

China. Yunnan Province, Malipo County, Mount Laoshan, in thin forests on the slopes of limestone hills, 22°59.08'N, 104°50.48'E, 1160 m a.s.l., 14 October 2019, *Q. Fan 17570* (holotype: SYS; isotypes: IBSC, SYS). (Figs 2, 3)

##### Diagnosis.

This species is similar to *E. malipoensis* and *E. serrata*, but differs from them in its leaf shape, indumentum on the lower leaf surfaces, longer petioles, much fewer flowers on the panicle, larger flowers, and other traits.

##### Description.

Evergreen small tree, 4–10 m tall, much branched; stems 8–25 cm in diameter; branchlets grey-white, terete, glabrous, 6–10 mm in diameter. Leaves spirally inserted on branches and often crowded at tips of branchlets; petioles 2–5 cm long, glabrous; stipules elliptic or ovate-lanceolate, 1–3 × 0.5–1 cm, glabrous, caducous; leaf blades oblong or broad elliptic, 20–40 × 7–12 cm, thickly coriaceous, glabrous, midrib elevated on both surfaces, secondary veins 21–30 pairs, arching slightly and often dichotomous before reaching the margin, elevated on both surfaces margin serrate, apex acute or cuspidate, base cuneate, gradually tapering to the petiole. Inﬂorescence in terminal panicles, 15- to 30-flowered, 8–15 cm long, 6–10 cm in diameter, with 6–10 lateral racemes, the lowermost laterals in the axils of reduced leaves (often almost entirely consisting of the stipules only), upper ones in axils of bracts, lateral racemes sometimes branched in the lower part of the inflorescence; peduncle and pedicels densely yellow-brown tomentose; bracts ovate-triangular, 1–1.5 cm long, abaxially tomentose, adaxially glabrous or sparsely pubescent; bracteoles subulate or triangular, 3–8 mm long, abaxially densely tomentose, adaxially pubescent. Flowers 2.5–3 cm in diameter. Hypanthium obconical, 4–6 × 5–7 mm, abaxially densely yellow-brown tomentose, 5-lobed, the calyx lobes ovate, 3–5 × 2–4 mm, abaxially densely tomentose, adaxially glabrous; petals white, obovate or rotund, 6–9 × 5–10 mm, shortly clawed, glabrous, margin crisped or irregularly crenulate, apex retuse; stamens 20; flaments 3–6 mm long, glabrous; anthers 1–2 mm long; ovary semi-inferior, the free apex densely villous, ovoid, 2–3 mm across, 3–5-loculed, with 2 ovules per locule; styles 3–5, densely villous, 5–7 mm long, connate at base or fused from base to middle; ovules ovoid or ellipsoid, c. 1 mm across. Pome yellow at maturity, subglobose, 2.5–3.5 cm in diameter, glabrescent, crowned by the persistent calyx lobes forming an apical beak; pericarpium fleshy, ca. 3 mm thick. Seeds (1-) 2 per fruit.

##### Phenology.

Flowering from September to October, fruiting from November to December.

##### Etymology.

The specifc epithet refers to Laoshan Mountain, the locality of the type collection.

##### Distribution and habitat.

*Eriobotrya laoshanica* is currently known only from two localities in Laoshan Natural Reserve, Malipo County, southeastern Yunnan, China. Here, the species is distributed in thin forests on the slopes of limestone hills at altitudes of 1100–1358 m a.s.l. The common associated tree species include *Aucuba chinensis*, *Caryodaphnopsis tonkinensis*, *Ficus semicordata*, *Firmiana* sp., *Garcinia paucinervis*, *Machilus* sp. and *Syzygium claviflorum*.

##### Conservation status.

Only two populations were found with no more than 50 mature individuals in a total area of about 5 km^2^. It’s about 6.5 km away between the two populations. The wood of this species is very suitable for firewood. During the expedition in 2019, we found that at least two big trees about 15 cm in diameter were felled by the local villagers. Thus the species could be considered as CR (Critically Endangered) status according to IUCN Red List criteria (B2ab(v); IUCN 2019).

##### Note.

**Figure 4.**
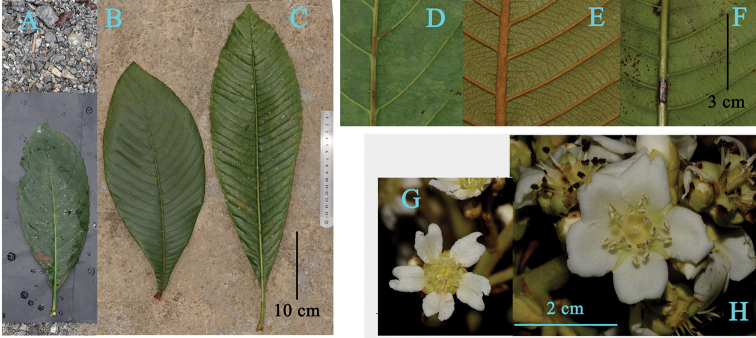
Morphological comparisons amongst *Eriobotrya laoshanica*, *E. malipoensis* and *E. serrata*. **A–C** leaves of *E. serrata* (**A**), *E. malipoensis* (**B**) and *E. laoshanica* (**C**) **D–F** abaxial leaf surface of *E. serrata* (**D**), *E. malipoensis* (**E**) and *E. laoshanica* (**F**) **G, H** flowers of *E. malipoensis* (**G**) and *E. laoshanica* (**H**). Photos taken by Qiang Fan.

The closest relative of *Eriobotrya laoshanica* on morphological grounds could be *E. malipoensis* Kuan, which usually coexists with the new species. They shared several characteristics, e.g., the long thick-coriaceous leaves that are up to 40 cm long; styles 3–5; and the subglobose fruits. The new species can be distinguished from *E. malipoensis*, however, by its longer petioles (2–5 vs. 0.5–1 cm); indumentum on the lower leaf surfaces (densely tomentose vs. glabrous); much fewer flowers (15- to 30-flowered vs. 50- to 100-flowered) on the panicle; larger flowers (2.5–3 vs. 1.5–2 cm in diameter); and non-angulated (vs. angulated) young fruits. *E. laoshanica* also has some resemblance to *E. serrata* Vidal but differs in its thicker leaves; leaf shape (oblong to broad elliptic vs. obovate to oblanceolate); more lateral veins (21–30 vs. 10–16 pairs); and larger fruits (2.5–3.5 vs. 1.5–1.8 cm in diameter) (Table 1 and Fig. 4).

To distinguish these species of *Eriobotrya* flowering in autumn and winter (from September to February) in China, an identification key is provided (based on Zhang et al. 1990; Gu and Spongberg 2003; Ding et al. 2015).

**Table 1.** Morphological comparisons amongst *Eriobotrya laoshanica*, *E. malipoensis* and *E. serrata*.

Characters	*E. laoshanica*	*E. malipoensis*	*E. serrata*
Leaf shape and size	oblong or broad elliptic, 20–40 × 7–12 cm	oblong or oblong-obovate, 30–40 × 10–15 cm	obovate or oblanceolate, 9–23 × 3.5–13 cm
Texture of leaves	thickly coriaceous	thickly coriaceous	thinly coriaceous
Indumentum on the lower leaf surfaces	glabrous	densely rusty tomentose	tomentose when young, glabrescent
Petiole length	2–5 cm	0.5–1 cm	1.5–3 cm
Lateral veins	21–30 pairs	20–25 pairs	10–16 pairs
Inﬂorescences	with reduced leaves, 15- to 30-flowered	without reduced leaves, 50- to 100-flowered	without reduced leaves, 30- to 60-flowered
Flower size (diameter)	2.5–3 cm	1.5–2 cm	1–2 cm
Fruit shape and size (diameter)	subglobose, 2.5–3.5 cm	pyriform, 2–3.5 cm	ovoid or pyriform, 1.5–1.8 cm

##### Additional specimens examined (paratypes).

China. Yunnan: Malipo, Laoshan natural reserve, 22°58.66'N, 104°50.80'E, 1135 m a.s.l., 16 September 2015 (young fl.), *Q. Fan 13700* (SYS); the same locality, 16 September 2015 (young fl.), *Q. Fan 13701* (SYS); the same locality, 22°59.10'N, 104°50.64'E, 1140 m a.s.l., 30 November 2015 (young fr.), *Q. Fan 13887* (SYS); the same locality, 1140 m a.s.l., 30 November 2015 (fr.), *Q. Fan 13900* (SYS); the same locality, 1140 m a.s.l., 30 November 2015 (fr.), *Q. Fan 13901* (SYS); the same locality, 1160 m a.s.l., 26 September 2019 (young fl.), *Q. Fan 17540* (SYS); the same locality, 1358 m a.s.l., 26 September 2019 (no fl. and no fr.), *Q. Fan 17543* (SYS).

### Key to *Eriobotrya* species flowering in autumn and winter in China

**Table:** 

1	Leaves abaxially glabrous or glabrescent	**2**
–	Leaves abaxially rusty or gray persistent tomentose	**3**
2	Leaves abaxially glabrous	***E. laoshanica***
–	Leaves abaxilly brownish yellow tomentose when young, glabrescent	***E. serrata***
3	Leaves abaxially rusty tomentose; leaf blade 30–40 cm long; lateral veins 22–25 pairs	***E. malipoensis***
–	Leaves abaxially gray tomentose; leaf blade 7–30 cm long; lateral veins 9–16 pairs	**4**
4	Leaf blade adaxially rugose; styles 5	***E. japonica***
–	Leaf blade adaxially not rugose; styles 2–4, rarely 5	**5**
5	Stipule subulate; inflorescences 8–12 cm long; pome 1.5–3 cm in diam	**E. × daduheensis**
–	Stipule ovate; inflorescences 6–10 cm long; pome 0.6–1 cm in diam	***E. prinoides***

## Supplementary Material

XML Treatment for Eriobotrya laoshanica
